# Comparison of the Amyloid Plaque Proteome in Down Syndrome, Early-Onset Alzheimer’s Disease and Late-Onset Alzheimer’s Disease

**DOI:** 10.21203/rs.3.rs-4469045/v1

**Published:** 2024-07-15

**Authors:** Mitchell Martá-Ariza, Dominique F Leitner, Evgeny Kanshin, Jianina Suazo, Ana Giusti Pedrosa, Manon Thierry, Edward B. Lee, Orrin Devinsky, Eleanor Drummond, Juan Fortea, Alberto Lleó, Beatrix Ueberheide, Thomas Wisniewski

**Affiliations:** New York University Grossman School of Medicine; New York University Grossman School of Medicine; New York University Grossman School of Medicine; New York University Grossman School of Medicine; New York University; New York University Grossman School of Medicine; University of Pennsylvania Perelman School of Medicine; New York University Grossman School of Medicine; The University of Sydney; Universitat Autònoma de Barcelona: Universitat Autonoma de Barcelona; Universitat Autònoma de Barcelona: Universitat Autonoma de Barcelona; New York University Grossman School of Medicine; New York University School of Medicine

**Keywords:** Down syndrome, Alzheimer’s disease, Proteomics, Amyloid-β, Neuropathology

## Abstract

**Background:**

Down syndrome (DS) is strongly associated with Alzheimer’s disease (AD), attributable to *APP* overexpression. DS exhibits Amyloid-β (Aβ) and Tau pathology similar to early-onset AD (EOAD) and late-onset AD (LOAD). The study aimed to evaluate the Aβ plaque proteome of DS, EOAD and LOAD.

**Methods:**

Using unbiased localized proteomics, we analyzed amyloid plaques and adjacent plaque-devoid tissue (‘non-plaque’) from post-mortem paraffin-embedded tissues in four cohorts (n = 20/group): DS (59.8 ± 4.99 y/o), EOAD (63 ± 4.07 y/o), LOAD (82.1 ± 6.37 y/o) and controls (66.4 ± 13.04). We assessed functional associations using Gene Ontology (GO) enrichment and protein interaction networks.

**Results:**

We identified differentially abundant Aβ plaque proteins vs. non-plaques (FDR < 5%, fold-change > 1.5) in DS (n = 132), EOAD (n = 192) and in LOAD (n = 128); there were 43 plaque-associated proteins shared between all groups. Positive correlations (*p* < 0.0001) were observed between plaque-associated proteins in DS and EOAD (R^2^ = 0.77), DS and LOAD (R^2^ = 0.73), and EOAD vs. LOAD (R^2^ = 0.67). Top Biological process (BP) GO terms (*p* < 0.0001) included lysosomal transport for DS, immune system regulation for EOAD, and lysosome organization for LOAD. Protein networks revealed a plaque enriched signature across all cohorts involving APP metabolism, immune response, and lysosomal functions. In DS, EOAD and LOAD non-plaque vs. control tissue, we identified 263, 269, and 301 differentially abundant proteins, including 65 altered non-plaque proteins across all cohorts. Differentially abundant non-plaque proteins in DS showed a significant (*p* < 0.0001) but weaker positive correlation with EOAD (R^2^ = 0.59) and LOAD (R^2^ = 0.33) compared to the stronger correlation between EOAD and LOAD (R^2^ = 0.79). The top BP GO term for all groups was chromatin remodeling (DS *p* = 0.0013, EOAD *p* = 5.79×10^− 9^, and LOAD *p* = 1.69×10^− 10^). Additional GO terms for DS included extracellular matrix (*p* = 0.0068), while EOAD and LOAD were associated with protein-DNA complexes and gene expression regulation (*p* < 0.0001).

**Conclusions:**

We found strong similarities among the Aβ plaque proteomes in individuals with DS, EOAD and LOAD, and a robust association between the plaque proteomes and lysosomal and immune-related pathways. Further, non-plaque proteomes highlighted altered pathways related to chromatin structure and extracellular matrix (ECM), the latter particularly associated with DS. We identified novel Aβ plaque proteins, which may serve as biomarkers or therapeutic targets.

## Introduction

Down syndrome (DS) is the most prevalent chromosomal abnormality, characterized by the partial or complete triplication of chromosome 21 (Hsa21) (1, 2). DS is strongly associated with Alzheimer’s disease (AD) due to the presence/duplication of the amyloid-β precursor protein (*APP*) gene in Hsa21 (3–5). Hsa21 also contains other genes of interest for AD, such as S100β (associated with astrocytes), *DYRK1A* (encodes for a kinase that phosphorylates Tau), *SOD1* and *BACE2* (related to oxidative stress) (6–10), which may play a role in AD in addition to *APP*. By age 40, virtually all individuals with DS exhibit AD pathological hallmarks, including extracellular amyloid-β (Aβ) accumulation and neurofibrillary tangles formed by hyperphosphorylated Tau (11–13). Brain atrophy and elevated cerebrospinal fluid and plasma levels of Aβ42 and neurofilament light, respectively, have been observed in people with DS (14). These neuropathological features are qualitatively similar to other AD forms, such as early and late-onset AD (14, 15).

Earlier investigations and most recent findings suggests that AD neuropathology extends beyond Aβ and Tau proteins (12, 16), implicating hundreds of associated proteins in biological dysfunctions such as synaptic transmission, immune response, mitochondrial metabolism, and oxidative stress (17–19). Proteomic comparisons between DS and early-onset AD (EOAD) Aβ plaques reveal common proteins enriched in both conditions, although differences in protein abundance have been observed (12). Despite recent progress, the molecular mechanisms of AD remain elusive, particularly regarding common pathophysiological mechanisms across AD subtypes and the specifics of AD neuropathogenesis in DS. Individuals with DS develop AD neuropathology earlier than the general AD population, with Aβ and Tau accumulation patterns mirroring those in AD (20). However, the extent to which the protein composition in DS pathological lesions aligns with other AD subtypes remains uncertain (21). Identifying gene-phenotype associations in DS is also challenging due to multiple triplicated genes (15). Given these complexities, DS is particularly relevant as an AD model, due to the universal prevalence of DS with AD pathology with increasing age, compared to the other dominant inherited forms of Alzheimer’s and the more homogeneous, age-dependent pathology compared to sporadic AD (15, 22–24).

In light of these findings, this study aimed to characterize the proteomic differences among AD subtypes. In particular, we examined the Aβ plaque proteome in DS, EOAD, and LOAD, expanding on prior DS and EOAD comparisons (12). Our analysis revealed a substantial similarity of proteins enriched in Aβ plaques across all experimental groups, providing new evidence about the Aβ plaque protein composition of individuals with DS in direct comparison with EOAD and LOAD. The proteomes also shared functional associations, thus revealing a consistent plaque protein signature in DS, EOAD and LOAD. Despite the enrichment of similar plaque proteins in all cohorts, we observed subtle differences in the proteome composition, characterized by variations in protein abundance in each group. Corresponding observations were made in the proteomic composition of DS, EOAD and LOAD non-plaque tissue compared to controls. These insights may contribute to identifying novel therapeutic targets or biomarkers tailored to the specific features of different AD subtypes.

## Methods

### Human brain tissue

*Post-mortem* formalin fixed and paraffin embedded (FFPE) brain tissues from DS, EOAD, LOAD and cognitive normal age-matched controls (n = 20 brain cases for each cohort) were obtained from the National Institutes of Health NeuroBioBank (Maryland and Mt. Sinai brain banks), UK Brain Bank Network (South West Dementia brain bank), IDIBAPS Biobank from Barcelona, University of Pennsylvania and NYU Grossman School of Medicine, including autopsy tissues from NYU Alzheimer’s Disease Research Center (ADRC), Center for Biospecimen Research and Development (CBRD)/Department of Pathology and the North American SUDEP Registry (NASR) at NYU Comprehensive Epilepsy Center (CEC). FFPE tissue blocks containing hippocampus and surrounding entorhinal and temporal cortex were used for the present study as it contains a high amount of amyloid pathology. The cases were assessed by the brain repositories to confirm advanced AD, by ABC neuropathological score (25–27). Further details about the cases are included in Table 1 and detailed case history is provided in **Supp. Table. 1**. Cases lacking information about α-synuclein and TDP-43 were stained by CBRD and assessed in the laboratory. Inclusion criteria for all cases included tissue formalin fixation below 3 years. We tolerated cases with TDP-43 (DS = 2, EOAD = 2, LOAD = 1) or α-synuclein (DS = 7, EOAD = 2, LOAD = 1) inclusions in order to increase the number of cases, as these co-pathologies are common in the elderly population and did not affect our comparative proteomics analysis. We performed one-way ANOVA analysis followed by *post hoc* Tukey’s multiple comparison test to determine significant age differences among the cohorts evaluated and multiple variable linear regression to determine what clinical variables may have influenced the proteomics results.

### APOE genotyping

*APOE* genotyping was conducted for the cases where this information was not provided by the brain banks, following a previously established protocol (12). Briefly, DNA extraction from FFPE tissue scrolls was performed using the QIAamp DNA FFPE Advanced UNG Kit (Qiagen, cat. 56704) as indicated by the manufacturer. Two endpoint PCRs were carried out using custom primers (Forward primer 5’ AGGCCTACAAATCGGAACTGG 3’; reverse primer 5’ CCTGTTCCACCAGGGGC 3’; Sigma). After the initial PCR, DNA purification from the agarose gel was accomplished using the QIAquick Gel Extraction Kit (Qiagen, cat. 28704), following the manufacturer's protocol. Subsequently, the gel-purified DNA was used for the second endpoint PCR, followed by Sanger sequencing and sequence analysis using SnapGene 5.3.1 software.

### Immunohistochemistry for Aβ and pTau

FFPE 8 μm tissue sections that contain the hippocampus and adjacent temporal cortex were collected on glass slides. Sections underwent chromogenic immunohistochemistry for total Aβ (Aβ 17–24 clone 4G8, 1:1000, BioLegend, cat. 800710) and Tau pathology (PHF-1, 1:200, in house developed mouse monoclonal antibody provided by Dr. Peter Davies, Albert Einstein University, NY, USA (28)). Sections were deparaffinized and rehydrated through a brief series of xylene and ethanol washes. Antigen retrieval methods performed include a 7-minute treatment of 88% formic acid followed by heat-induced citrate buffer treatment (10mM sodium citrate, 0.05% Tween-20; pH 6). Endogenous peroxidase was quenched with 0.3% H_2_O_2_ solution for 20 minutes. Sections were blocked with 10% normal goat serum, proceeded by an overnight incubation with the primary antibody diluted in 4% normal goat serum. Sections were incubated for 1 hour at room temperature with the appropriate secondary antibody (biotinylated HRP mouse IgG, 1:1000, Vector, cat. BA-2000). Staining signal was amplified using VECTASTAIN Avidin-Biotin Complex (ABC) kit (Vector, cat. PK6100) for 30 min. The chromogen DAB was used to visualize the pathology. Sections were counterstained with hematoxylin and coverslipped using the appropriate mounting media. Aβ and Tau quantities were quantified from whole slide scans at 20X magnification using a Leica Aperio Versa 8 microscope. Five regions of interest (ROIs) in the temporal cortex and hippocampus (CA1, CA2, CA3) were used to calculate the percent positive pixel area. We used a custom macro based on the ‘Positive Pixel Count’ algorithm in ImageScope v.12.4.3.5008, with a modification to the ‘Color saturation threshold’ = 0 and the ‘Upper limit of intensity for weak-positive pixels’ (Iwp high) = 190. Statistical differences between experimental groups were evaluated using one-way ANOVA followed by Tukey’s multiple comparisons test in GraphPad Prism v 9.5.1. Data is shown as mean ± standard error of the mean (SEM).

**Figure 1:**
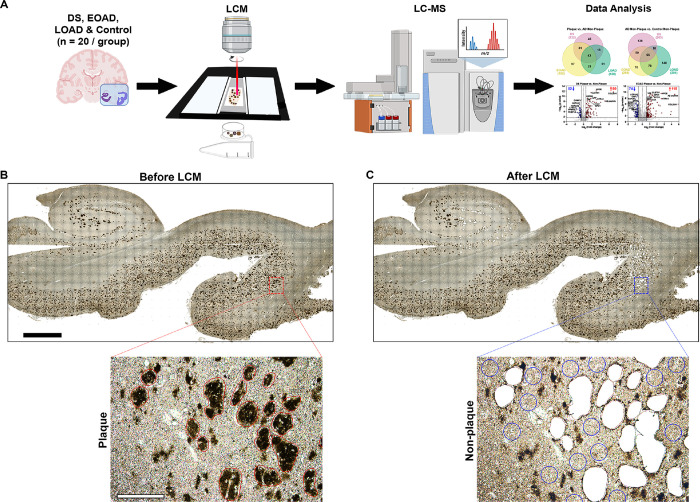
Schematic of the localized proteomics protocol. **A.** Laser-capture microdissection of 2 mm^2^ total area of amyloid-β plaques from hippocampus and adjacent temporal cortex from FFPE autopsy brain tissue from control, DS, EOAD and LOAD (n=20 cases/experimental group). Amyloid plaque proteins were quantified by label-free mass spectrometry and posteriorly analyzed. **B-C.** Microphotographs of a typical brain tissue section immunolabeled against Aβ illustrate the precise microdissection of amyloid plaques before (**B**) and after LCM (**C**). 2 mm (black bar, top) and 200 μm (white bar, bottom).

Unbiased localized proteomics was performed using the method outlined in Fig. 1A. FFPE tissues were cut into 8 μm sections from autopsy hippocampal and adjacent entorhinal and temporal cortex tissues onto laser-capture microdissection (LCM) compatible PET membrane slides (Leica, cat. 11505151). Amyloid-β deposits were visualized by immunohistochemistry using the pan-Aβ 4G8 antibody (1:1000, BioLegend, cat. 800710), by using the chromogen 3,3-diaminobenzidine (DAB, Thermo Scientific, cat. 34065) reaction. Classic cored, neuritic and dense Aβ plaques were targeted (not diffuse or cotton wool plaques) for a more homogeneous analysis, using LCM to dissect a total area of 2 mm^2^ and the same area for neighboring non-plaque tissue (Fig. 1B–C), at 10X magnification with a LMD6500 microscope equipped with a UV laser (Leica). We avoided diffuse amyloid aggregates in all the cases used to maintain samples consistency. Microdissected samples were centrifuged for 2 min at 14,000 g and stored at − 80°C. We also microdissected adjacent tissue free of plaques from the same microscopic field of views that contained microdissected amyloid plaques, but at a sufficient distance from plaques to ensure that plaque-associated tissue was not collected (Fig. 1C). These samples are henceforth referred to as ‘non-plaque’. In addition, analogous non-plaque tissue from control cases was selected from matching hippocampal and temporal cortex regions as those used in DS, EOAD and LOAD, denoted as ‘Control non-plaque’. The schematic diagrams for the figure were generated using BioRender.com.

### Label-free quantitative mass spectrometry (MS) proteomics

The extraction and digestion of proteins from Laser Capture Microdissection (LCM) excised plaque and non-plaque tissue samples were performed using the SPEED sample prep workflow (29). Briefly, tissue sections were incubated in 10 μl of LC-MS grade formic acid (FA) for 5 minutes at 73°C. The FA was then neutralized by a 10-fold dilution with 2M TRIS containing 10 mM Tris (2-carboxyethyl) phosphine (TCEP) and 20 mM chloroacetic acid (CAA), followed by an incubation at 90°C for 1 h. For enzymatic digestion, samples were diluted six-fold with water containing 0.2 μg of sequencing-grade trypsin. Digestion was carried out overnight at 37°C and halted by acidification to 2% TFA.

Liquid chromatography-tandem mass spectrometry (LC-MS/MS) was performed online on an Evosep One LC using a Dr. Maisch ReproSil-Pur 120 C18 AQ analytical column (1.9-μm bead, 150 μm ID, 15 cm long). Peptides were gradient eluted from the column directly into an Orbitrap HF-X mass spectrometer using the 88-minute extended Evosep method (SPD15) at a flow rate of 220 nl/min. The mass spectrometer was operated in data-independent acquisition (DIA) mode, acquiring MS/MS fragmentation across 22 m/z windows after every MS full-scan event.

High-resolution full MS spectra were acquired with a resolution of 120,000, an Automatic Gain Control (AGC) target of 3e6, a maximum ion injection time of 60 ms, and a scan range of 350 to 165 m/z. Following each full MS scan, 22 data-independent higher-energy collisional dissociation (HCD) MS/MS scans were acquired at a resolution of 30,000, an AGC target of 3e6, and a stepped normalized collision energy (NCE) of 22.5, 25, and 27.5.

### Proteomics computational analysis

The analysis of the MS data was conducted utilizing the Spectronaut^®^ software (https://biognosys.com/shop/spectronaut), searching in direct-DIA mode (w/o experimental spectral library) against the Homo Sapiens UniProt database (http://www.uniprot.org/) concatenated with a list of common lab contaminants. The integrated search engine, Pulsar, was employed for the database search. The enzyme specificity was configured to trypsin, allowing for up to two missed cleavages during the search process. The search also included oxidation of methionine as a variable modification, and carbamidomethylation of cysteines as a fixed modification. The false discovery rate (FDR) for identification of peptide, protein, and site was limited to 1%. Quantification was performed on the MS/MS level, utilizing the three most intense fragment ions per precursor. For subsequent data analysis, the Perseus (30), R environment (http://www.r-project.org/), or Prism GraphPad were used for statistical computing and graphics.

### Proteomics statistical analyses

The protein expression matrix (n = 2080) was filtered to remove common laboratory contaminants, non-human proteins and those proteins observed in less than half of all the 4 groups evaluated (n = 1995). For principal component analysis (PCA), missing values were imputed from the normal distribution with a width of 0.3 and a downshift of 1.8 (relative to measured protein intensity distribution) using Perseus v 1.6.14.0 (30). We performed paired *t*-tests to evaluate the amyloid plaques enrichment in relation to the non-plaque tissue adjacent to the amyloid plaques. In addition, we performed unpaired *t*-tests to compare the protein enrichment of non-plaques from DS, EOAD and LOAD compared to control tissue samples. Proteins were deemed significantly altered if they had a false discovery rate (FDR) below 5% (permutation-based FDR with 250 data randomizations). We further filtered the significant proteins based on the fold change (FC) difference > 1.5 fold between the groups. The proteins of interest common to each pairwise comparison from ‘plaques vs. non-plaque’ and ‘non-plaque vs. control non-plaque’ tissue were evaluated by Venn diagrams generated from InteractiVenn (31). Pearson’s correlation analysis between DS, EOAD and LOAD differentially abundant proteins identified in the pairwise comparisons were evaluated using GraphPad Prism v 9.5.1. For this analysis, we considered proteins that were significantly altered in at least one of the groups and had a FC > 1.5, on a given correlation.

### Mapping protein-coding genes to the Hsa21

Genes coding for the proteins identified in the study were mapped to their respective chromosomes in R using the function ‘mapIds’ from the *Annotation DBI* package v 1.62.2 with the genome-wide annotation for human, org.Hs.eg.db v 3.17.0. Chromosome 21 (*Homo sapiens* autosome 21, or Hsa21) location for each gene was determined using the UCSC Human Genome Browser (32).

### Gene Ontology functional annotation

Gene Ontology (GO) enrichment analysis was performed in R using the package *clusterProfiler* v 4.8.2, with the genome-wide annotation for human, org.Hs.eg.db v 3.17.0. GO terms were filtered to an FDR < 0.05 using the Benjamini-Hochberg method (33). Isoform labels were excluded from Uniprot accession IDs for GO functional annotation. Duplicate proteins were removed, and the resulting list comprising 1980 proteins lacking isoforms was utilized as the background dataset. Functional annotation was focused on GO biological process (GO BP) and GO cellular component (GO CC). Heavily redundant GO terms were reduced using the ‘simplify’ function from clusterProfiler, with a cutoff of 0.7. Top 10 significantly enriched GO terms for highly abundant proteins in ‘plaques vs. non-plaque’ and ‘non-plaque vs. control non-plaque’ for each experimental group were selected using the adjusted *p* value (−Log_10_ adj. *p*-value) and compared using heatmaps generated in GraphPad Prism.

### Protein-protein interaction networks

Protein-protein interaction (PPI) networks were made in Cytoscape v 3.10.0 using ‘STRING: protein query’ (STRING v 11.5 database (34)) with a (high) confidence score of 0.7. Networks reflect functional and physical protein associations for the differentially abundant proteins in DS, EOAD and LOAD. Node size of the networks indicate the adjusted *p* value (−log_10_ [*p*-value]) from the *t*-tests and node color indicates fold-change (log_2_ [FC]). Disconnected nodes were not depicted in the final network. Dotted-line colored boxes highlight proteins clustered by function similarity.

### Comparison with previous AD proteomics studies in human brain

Our data was compared to previous proteomic studies using the NeuroPro database (v1.12; https://neuropro.biomedical.hosting/) (35). NeuroPro is a combined analysis of differentially enriched proteins found in human AD brain tissues identified in 38 published proteomics studies (at the time of use for this study, February 2024). NeuroPro database was filtered to include only proteins found in advanced AD proteomics studies (AD and AD/C). Alternatively, we applied a second filter to advanced AD to include proteomics studies in ‘plaques’ only. Protein lists obtained after filtering the NeuroPro database were manually curated to address current ‘obsolete deleted’, ‘merged’ or ‘demerged’ UniProt accession IDs. We performed a manual curation of NeuroPro protein lists to provide an accurate comparison between the proteins identified in previous proteomics studies and our present study. The UniProt accession IDs and gene IDs from the proteins we identified in the current study were matched to the IDs from the NeuroPro to identify proteins that have not been previously associated with human AD and amyloid plaque proteomics.

Additionally, as the NeuroPro database does not include DS proteomics data, we compared our current DS plaques dataset with our previous DS plaque proteomics study (12). We identified the common proteins using the whole data matrix of both studies, by comparing the Uniprot Accession ID and the Gene ID, to account for any identifier differences. Then, we identified the significantly altered proteins on each study; for our dataset, we defined significantly altered proteins by FDR ≤ 5% and a fold change ≥ 1.5. In our previous study, significantly altered proteins were defined by *p* < 0.05 and a fold change ≥ 1.5. For the comparison, we included the significantly abundant and significantly decreased plaque proteins. We evaluated common significant proteins from the datasets using Venn diagrams generated from InteractiVenn (31). In addition, we performed Pearson’s correlation analysis between datasets using GraphPad Prism v 9.5.1. For the correlation analysis, we considered proteins that were significantly altered in at least one of the datasets.

## Results

### Amyloid-β and Tau pathologies are significantly increased in DS

AD pathology was assessed using the Braak and Thal staging or equivalent ABC score, for all cases used for proteomics analysis (Table 1, detailed case history in **Supp.** Table 1). Age was significantly different (*p* < 0.0001) in LOAD cohort in comparison to the other experimental groups. However, we included eight controls ≤ 65 years old and the remaining 12 cases ≥ 65 to compensate for the age gap between early and late onset forms of AD (**Supp.** Table 1). In addition, multiple variable linear regression analysis showed that age (*p* = 0.97) and sex (*p* = 0.45) did not contribute significantly to the differences observed in the proteomics analysis (**Supp.** Table 2).

Assessment of the distribution of Aβ and Tau pathology in all cases showed that Aβ levels in hippocampal and temporal regions were similar in DS and EOAD. However, Aβ quantities in DS were significantly higher (*p* = 0.013) compared to LOAD (**Supp.** Figure 1C). PHF-1 immunoreactive Tau pathology was significantly higher in DS compared to EOAD and LOAD (*p* = 0.0002 and *p* < 0.0001, respectively) (**Supp.** Figure 1D). Aβ and Tau pathology were not significantly different between EOAD and LOAD (**Supp.** Figure 1C-D). These results suggest an exacerbated Aβ and Tau pathology in DS despite the advanced stage of AD for all the cases in the cohorts evaluated.

### Protein abundance in amyloid plaques and non-plaque tissue varies across DS, EOAD and LOAD

#### Aβ Plaques Pairwise Comparisons

Protein differential expression in Aβ plaques and adjacent AD non-plaque tissue was evaluated using LFQ-MS in the microdissected hippocampus and temporal cortex (Fig. 1). LFQ-MS identified 1995 proteins (**Supp. Tables 3–4**), detected in at least 50% of the cases in any of the groups. PCA showed minimal segregation by groups (DS, EOAD, LOAD or control) or by sample type (plaques and non-plaque tissue).

**Figure 2:**
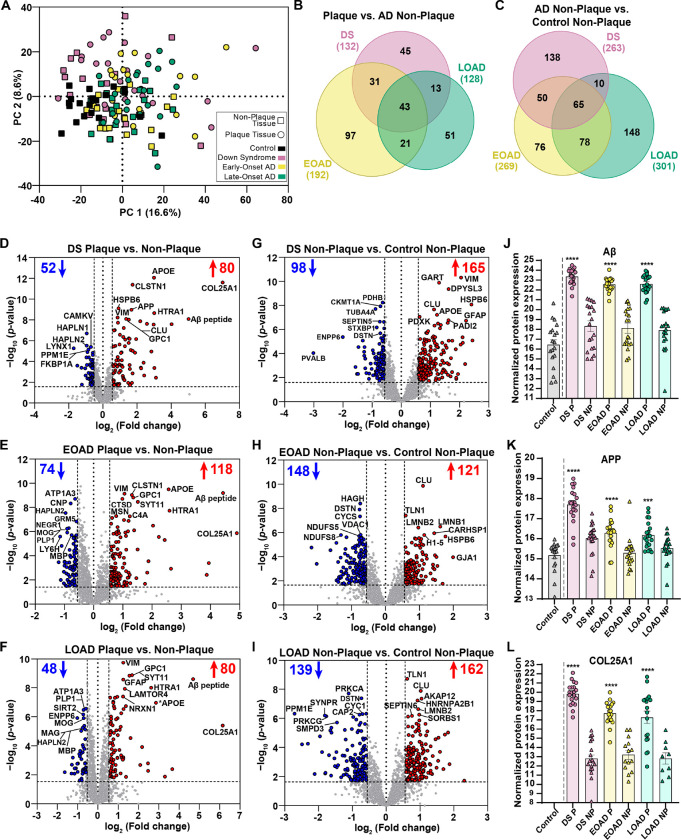
Principal component analysis (PCA) and differential protein expression in Aβ plaques and non-plaque tissue. **A.** PCA shows the distribution of the n=20 cases per each experimental group, with minimal segregation. **B.** Venn diagram of differentially abundant Aβ plaque proteins shows 43 common proteins for all the AD subtypes evaluated, 45 for DS, 97 for EOAD and 51 for LOAD. **C.** Venn diagram of differentially abundant non-plaque proteins depicts 138 proteins in DS, 76 proteins in EOAD, 148 proteins in LOAD, and 65 common proteins for all AD subtypes. **D-F.** Volcano plots indicate differentially expressed proteins (enriched in red, decreased in blue) in Aβ plaques compared to non-plaque tissue in DS (132 proteins, **D)**, EOAD (192 proteins, **E)** and LOAD (128 proteins, **F)**. **G-I**. Volcano plots depict differentially expressed proteins in DS non-plaque tissue compared to controls (263 proteins, **G**), EOAD non-plaques (269 proteins, **H**) and LOAD non-plaques (301 proteins, **I**). (**J-L**). Normalized protein expression obtained from the label-free quantitative mass spectrometry proteomics of Aβ peptide (**J**), APP protein (**K**) and COL25A1 (**L**). Significance was determined using a student’s two-tailed *t* test (FDR < 5%, fold-change > 1.5). *P* values are indicated based on the pairwise comparisons. *** *p*<0.001, *** *P*<0.0001. Error bars indicate standard error of the mean (SEM). Significant pairwise comparisons are indicated for those analyses that were performed, controls are shown as reference.

We identified 132 differentially abundant proteins in DS Aβ plaques compared to DS non-plaque tissue (Fig. 2B, D), 192 proteins in EOAD plaques vs. EOAD non-plaques (Fig. 2B, E) and 128 proteins in LOAD plaques vs. LOAD non-plaque tissue (FDR ≤ 5%, FC ≥ 1.5) (Fig. 2B, F). From these sets of proteins, 43 were shared between the three cohorts. We found 45 proteins with differential enrichment in plaques in DS, 97 proteins in EOAD and 51 proteins in LOAD (Fig. 2B), indicating that enrichment of some proteins in Aβ plaques is variable in each experimental group. We observed a consistent enrichment of AD associated proteins such as the Aβ specific peptide LVFFAEDVGSNK (sequence corresponds to amino acids 17–28 of Aβ, Fig. 2D–F, J) and other previously detected amyloid plaque proteins such as HTRA1, GPC1, VIM, APOE, CLSTN1 and SYT11 within the top 10 most significant proteins across groups (Table 2). As expected, APP was within the top 10 significantly abundant proteins in DS amyloid plaques (Fig. 2D), and was also significantly enriched in amyloid plaques in EOAD and LOAD (Fig. 2K). The plaque protein COL25A1 (Collagen alpha-1(XXV) chain, also known as CLAC-P) was the most abundant protein in amyloid plaques in all experimental groups, showing more enrichment in plaques than the Aβ peptide (Fig. 2D–F, L). Interestingly, COL25A1 was below mass spectrometry detection threshold in all control tissues (Fig. 2L), suggesting that this protein is highly correlated to Aβ plaque pathology. COL25A1 was increased 129.5-fold in DS, 29.9-fold in EOAD and 71-fold in LOAD (Table 2). In addition, COL25A1 was within the top 10 significant proteins only in DS (Table 2). Hyaluronan and proteoglycan link protein 2 (HAPLN2, also known as Bral1) was within the most significant proteins decreased in plaques in the three cohorts studied. In addition, we observed decreased plaque-protein levels of oligodendrocyte proteins. MOG was significantly decreased in all groups, and MAG and MBP were significantly decreased in EOAD and LOAD amyloid plaques respectively (**Supp. Table 3**). MAG and MBP levels were also decreased in plaques in DS, although it did not meet our significance criteria. The glucose transport facilitator SLC2A3 (also known as GLUT3) was decreased in amyloid plaques in all groups, yet it was significant only in EOAD and LOAD (Table 2). Overall, we observed similar proteins altered in Aβ plaques in all groups evaluated. However, most of the proteins show different abundance levels in plaques of DS, EOAD and LOAD, accounting for the differences observed among groups.

#### AD Non-plaque Tissue Pairwise Comparisons

In addition, we identified 263 differentially expressed proteins in DS non-plaque tissue compared to control non-plaque tissue (Fig. 2C, G), 269 proteins in EOAD non-plaque tissue vs. control non-plaque tissue (Fig. 2C, H) and 301 significantly altered proteins in LOAD non-plaque tissue vs. control non-plaque tissue (Fig. 2C, I). We identified 65 altered non-plaque proteins compared to control tissue that were common between all cohorts evaluated (Fig. 2C). We also observed 138 proteins with differential enrichment levels in DS non-plaque tissue, 76 proteins in EOAD and 148 proteins in LOAD (Fig. 2C). Notably, we identified among the top 10 enriched proteins in DS non-plaque tissue CLU, VIM, HSPB6 and SYNM (**Supp. Table 5**), which we also found enriched in amyloid plaques in all disease groups. CLU was consistently enriched in non-plaque tissue in the three groups evaluated when compared to control tissue (**Supp. Table 5**). VIM and HSPB6 were also among the most enriched proteins in EOAD non-plaque tissue (**Supp. Table 5**). Conversely, we identified the actin-binding protein destrin (DSTN) as the only protein within the top 10 significantly decreased proteins in non-plaque tissue that was present in all the cohorts analyzed (**Supp. Table 5**). We also observed that parvalbumin (PVALB) was the most decreased protein in DS non-plaque tissue compared with controls (Fig. 2G), whereas the levels of PVALB in EOAD and LOAD where not significantly different from controls (**Supp. Table 5**). Our proteomics findings in non-plaque tissue showed that there were more differences in protein levels in non-plaque tissue between groups, in comparison to the more consistent protein levels in plaques, highlighting the largely similar plaque proteome between AD subtypes despite differences in basal, non-plaque protein expression.

#### Amyloid plaque proteomes of DS, EOAD and LOAD are highly correlated

**Figure 3:**
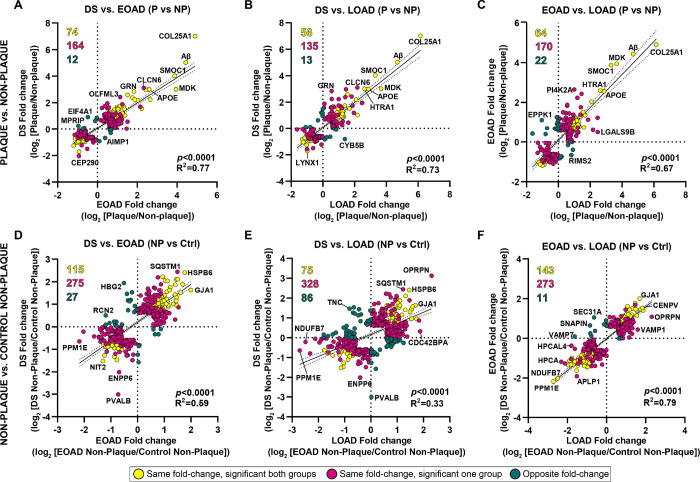
Correlation analyses of differentially abundant proteins in Aβ plaques and non-plaque tissue. (**A-C**) Correlation analyses for significant proteins in Aβ plaques vs non-plaque tissue and (**D-F**) DS, EOAD and LOAD non-plaque vs control non-plaque tissue. Yellow dots represent proteins changing in the same direction (highly abundant or less abundant proteins in both groups evaluated) and that are significant for both groups compared. Magenta dots represent proteins changing in the same direction, but are significant only in one of the groups evaluated. Green dots represent proteins changing in opposite direction (i.e., abundant in one group and less abundant in the other group evaluated). Numbers are colored to match the dots. Proteins were selected for the correlation analysis if they were significant at least in one of the groups compared and its fold change > 1.5. We observed a positive correlation between DS vs. EOAD (**A**) (*p*<0.0001, R^2^=0.77, (**B**) DS vs. LOAD (*p*<0.0001, R^2^=0.73) and (**C**) EOAD vs. LOAD (*p*<0.0001 R^2^=0.67). There is also a positive correlation when comparing non-plaque proteins in (**D**) DS vs. EOAD (*p*<0.0001 R^2^=0.59) and (**E**) DS vs. LOAD *p*<0.0001, R^2^=0.33). H. Correlation between EOAD and LOAD non-plaque proteins *p*<0.0001, R^2^=0.79).

We performed correlation analyses to compare the proteomes of Aβ plaques and non-plaque tissues in DS, EOAD and LOAD. Proteins included in the correlations were significant and FC > 1.5 at least in one of the groups evaluated. For amyloid plaques there was a positive correlation between DS and EOAD (R^2^ = 0.77, *p* < 0.0001). We observed 65.5% (164/250) of the proteins changing in the same direction (i.e., fold change for a protein is positive or negative in both groups), where 29.6% (74/250) of the proteins were significantly altered in DS and EOAD plaques (Fig. 3A). We only observed 4.8% (12/250) of the proteins changing in different directions (i.e., fold change for a protein is positive in one group and negative in the other) (Fig. 3A). DS and LOAD plaque proteomes also correlated positively (R^2^ = 0.73, *p* < 0.0001), with 66.2% (135/204) of the proteins with same fold change direction and 27.5% (56/204) of the proteins significantly altered in both groups (Fig. 3B). Similar to DS and EOAD, only 6.3% (13/204) of the proteins were changing in opposite direction (Fig. 3B). There was also a positive correlation between EOAD and LOAD differentially abundant plaque proteins (R^2^ = 0.67, p < 0.0001), similar to what we observed between DS vs. the AD subtypes evaluated. We identified 66.4% (234/256) of the proteins changing in the same direction, and 25% (64/256) of the proteins were significant in both groups (Fig. 3C). The proteins changing in opposite direction accounted for 8.6% (22/256) of the total (Fig. 3C). Our analysis shows high similarity among the proteins altered in Aβ plaques vs. non-plaques of DS, EOAD and LOAD, with the majority of the proteins changing in the same direction.

Correlation analyses of DS, EOAD and LOAD non-plaque differentially abundant proteins showed positive correlations between DS and EOAD (R^2^ = 0.59, *p* < 0.0001) and a weaker correlation between DS and LOAD (R^2^ = 0.33, *p* < 0.0001) (Fig. 3D–E). We observed 65.9% (275/417) of the proteins changing in the same direction in DS and EOAD Aβ plaques, where 27.6% (115/417) of the proteins were significantly altered in both groups. We observed 6.5% (27/417) of proteins changing in the opposite direction (Fig. 3D). Similarly, 67.1% (328/489) of the proteins in DS and LOAD were changing in the same direction (Fig. 3E). We observed that 15.3% (75/489) of the proteins were significant in both groups, whereas 17.6% (86/489) of proteins had opposite fold changes (Fig. 3E). Moreover, we observed a higher positive correlation between EOAD vs. LOAD non-plaque proteomes (R^2^ = 0.79, *p* < 0.0001), with 63.9% (273/427) of the proteins were changing in the same direction, with 33.5% (143/427) being also significant in both groups (Fig. 3F). Only 2.6% (11/427) of the proteins were changing in opposite directions (Fig. 3F). Overall, we observed a similar ‘amyloid plaques protein signature’ across the experimental groups. Nonetheless, correlations of the non-plaque tissue proteomes suggest a higher similarity between EOAD and LOAD differentially enriched proteins in comparison to DS.

#### Protein-coding genes present in Hsa21 do not lead to protein enrichment in Aβ plaques

**Figure 4:**
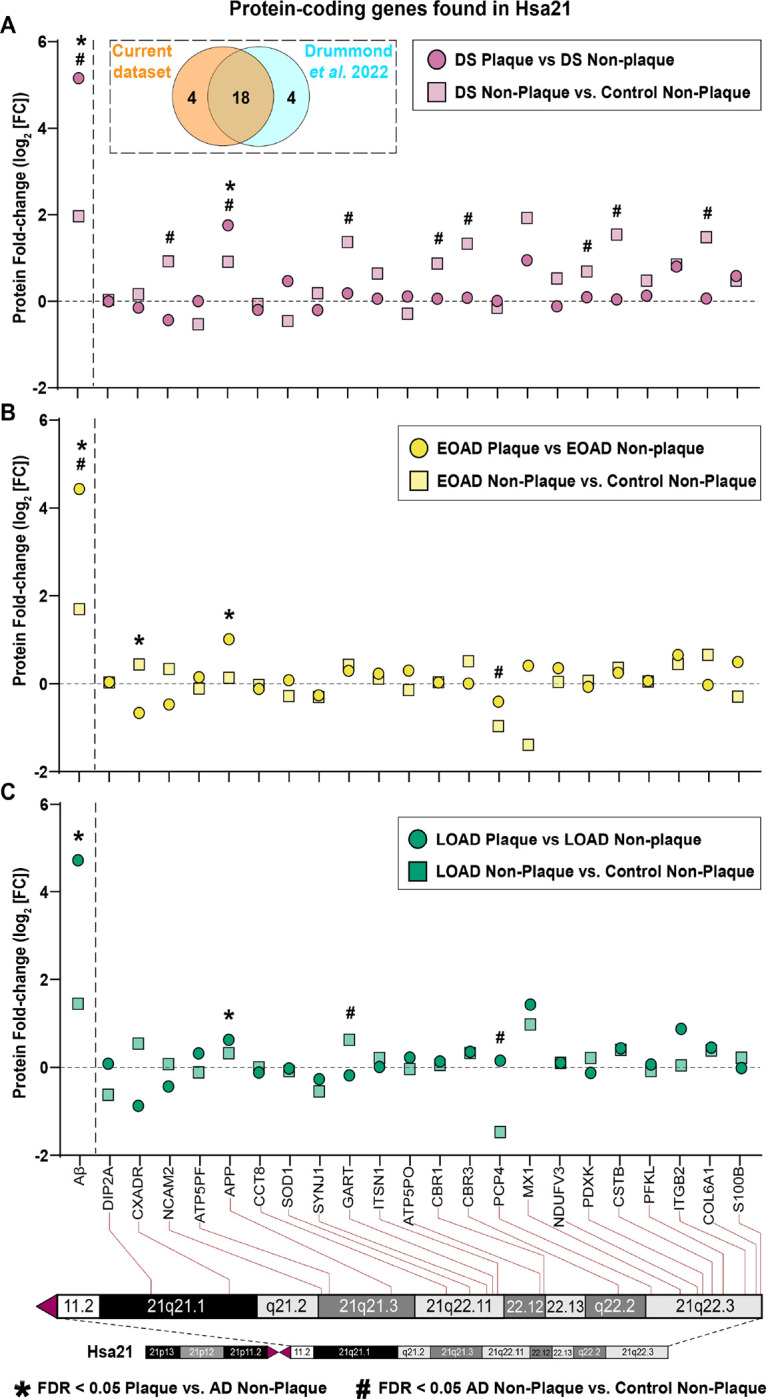
Mapping protein-coding genes to chromosome 21 (Hsa21). **A.** Dashed box contains Venn diagram of proteins from genes in Hsa21 identified in the current study vs. Drummond *et al*. 2022, (12). (**A-C**) The figure depicts fold change (Log_2_ FC) of the twenty-two Hsa21 genes whose corresponding protein products were found in Aβ plaques (circles) or neighboring non-plaque tissue (squares) in DS (**E**), EOAD (**F**) and LOAD (**G**). Paired two-tailed *t* tests (plaques vs. non-plaques) or unpaired two-tailed t tests (non-plaques vs. control) with permutation correction at a 5% FDR are indicated. Aβ peptide is shown as reference.

We mapped the protein-coding genes from chromosome 21 whose products were found in our proteomics analysis using the UCSC Human Genome Browser. From the 1995 proteins identified in this study, 22 come from Hsa21 (Fig. 4). We compared these proteins with the ones found in a previous DS plaque proteomics study (12), finding a total of 26 Hsa21 proteins identified between both studies. We observed 69.2% (18/26) of the proteins shared between the current and our previous study (Fig. 4). Among the proteins identified, APP was significantly altered in Aβ plaques in all cohorts (Fig. 4). APP was also significantly enriched in DS non-plaque tissue (FDR < 0.05, Fig. 4A). GART was significantly abundant in DS and LOAD non-plaques (Fig. 4A, C). NCAM2, CBR1, CBR3, PDXK, CSTB and COL6A1 were significantly enriched in DS non-plaque tissue (Fig. 4A). CXADR was differentially expressed in EOAD amyloid plaques (Fig. 4B), and PCP4 was differentially expressed in EOAD and LOAD non-plaque tissue (Fig. 4B–C). Despite the enrichment of some proteins in DS compared to control tissue, these results suggest that the triplication of the Hsa21 does not lead necessarily to enrichment of those gene products in Aβ plaques or surrounding tissue devoid of plaque pathology.

### Aβ plaque protein signature is related to APP processing, immunity and lysosomes

#### Aβ Plaques Functional Analyses

**Figure 5:**
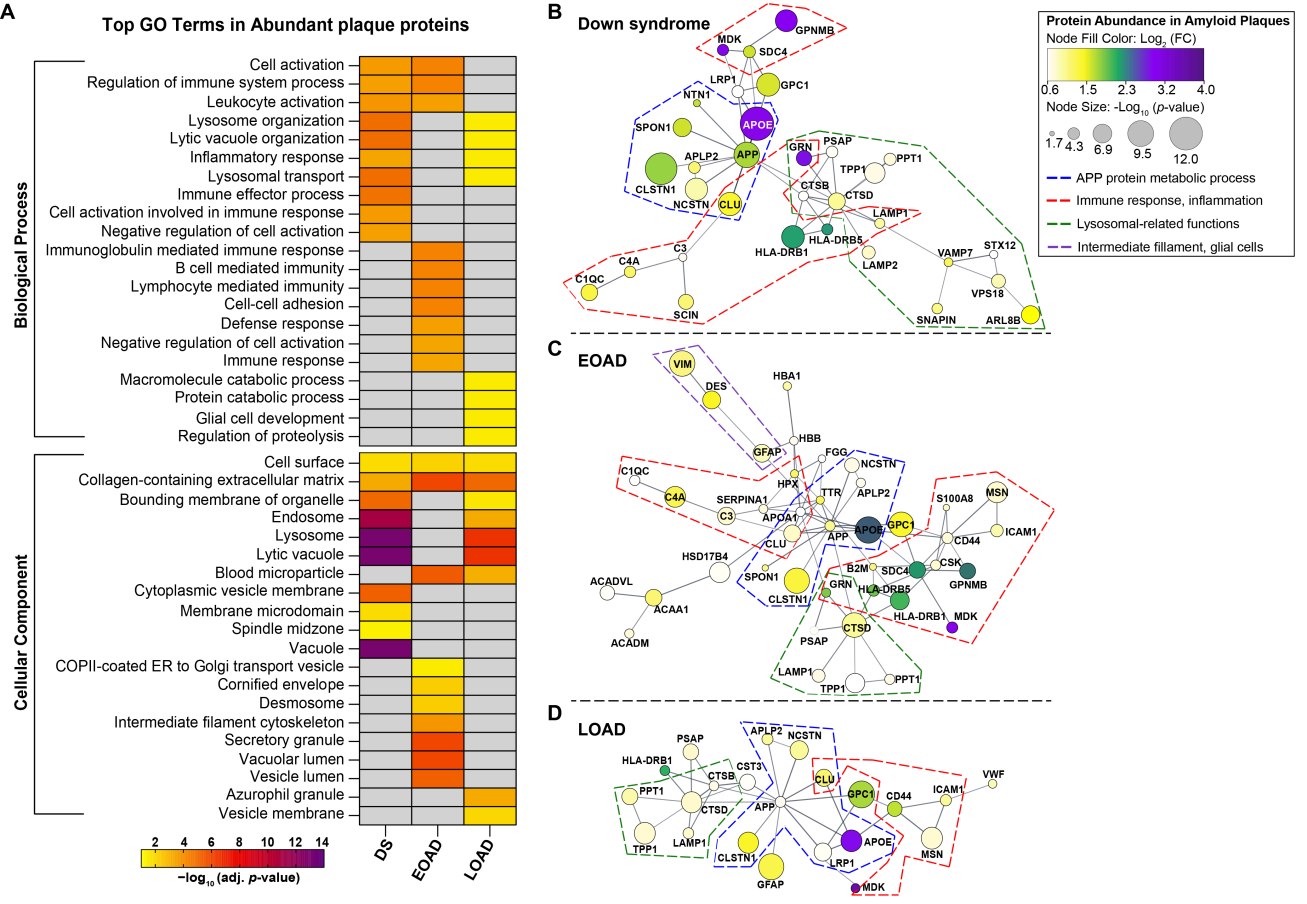
Gene ontology annotation and protein-protein interaction networks of significantly abundant proteins in Aβ plaques. **A.** GO terms heatmap depicts top 10 enriched BP and CC GO terms for significantly abundant Aβ plaque proteins in DS, EOAD and LOAD. Color indicates the adjusted *p*-value <0.05 (−Log_10_ [adj. *p*-value]). (**B-D**) Protein networks (PPI Enrichment *p*=1 × 10^−16^) show functional and physical amyloid plaques protein associations in DS (**B**), EOAD (**C**) and LOAD (**D**). Node color indicates fold-change (log_2_ [FC]) and node size depicts adjusted *p*-value (−log_10_ [*p*-value]) from the student’s two-tailed t test. Disconnected nodes are not shown in the network. Colored dotted lines highlight groups of proteins based on functions/pathways observed in the GO terms; Blue: APP protein metabolic process, Red: immune response and inflammation, Green: lysosomal-related functions, Purple: intermediate filament proteins, glial cells. GO terms annotation was performed using R package *clusterProfiler* v 4.8.2. PPI networks were created in Cytoscape v 3.10.0 using STRING database v 11.5.

We identified functional associations for the significantly abundant proteins in Aβ plaques and AD non-plaque tissue by performing ‘GO enrichment analysis’ (FDR < 0.05, **Supp. Tables 6–13**). Top enriched biological process (BP) GO terms in DS included lytic vacuole organization, lysosome organization and lysosomal transport (for the three terms, *p* = 1.29×10^− 5^, Fig. 5A, **Supp. Table 6**). We also identified terms cell activation (*p* = 0.00024), regulation of immune system process (*p* = 0.00027) and leukocyte activation (*p* = 0.00016), which were also observed in EOAD (Fig. 5A). For cellular component (CC) we identified as the top terms vacuole, lysosome, lytic vacuole (*p* = 9.56 ×10^− 14^), and endosome (*p* = 9.71 ×10^− 14^, Fig. 5A, **Supp. Table 10**), similarly as BP GO terms. In contrast, EOAD most enriched BP terms were regulation of immune system process, B cell mediated immunity, immunoglobulin mediated immune response and lymphocyte-mediated immunity (*p* = 4.33 ×10^− 5^, Fig. 5A, **Supp. Table 6**). Top CC GO terms in EOAD were secretory granule (*p* = 1.13 ×10^− 6^), vacuolar lumen and collagen-containing extracellular matrix (both *p* = 8.75 ×10^− 7^, Fig. 5A, **Supp. Table 10**). LOAD also showed BP GO terms related to lysosomes as observed in DS, yet with a lower significance. For instance, we identified lysosomal transport and organization and lytic vacuole organization (*p* = 0.0288 Fig. 5A, **Supp. Table 6**). CC GO terms included lysosome and lytic vacuoles (*p* = 2.47 × 10^− 7^), collagen-containing extracellular matrix (*p* = 9.41 ×10^− 6^) and endosome (*p* = 0.00063) (Fig. 5A, **Supp. Table 10**), highlighting functional similarities of plaque associated proteins between DS and LOAD.

We also evaluated the physical and functional protein interactions of significantly abundant proteins in Aβ plaques, using Cytoscape and the STRING database (Fig. 5B–D). The networks for amyloid plaque proteins for all the cohorts evaluated showed a significant degree of protein-protein interactions (PPI Enrichment *p* = 1 × 10^− 16^). We observed a consistent group of proteins in all forms of AD evaluated, which were grouped based on functional enrichment (Fig. 5B–D). For instance, we identified proteins related to APP and Aβ metabolism (APP, APOE, CLU, CLSTN1, NCSTN, APLP2, SPON1), immune response and inflammation (HLA-DRB1, HLA-DRB5, C1QC, C4A and C3 consistent in DS and EOAD; CD44, ICAM1 and MSN in EOAD and LOAD) and lysosomal-related functions (PPT1, TPP1, LAMP1, PSAP, CTSD). APOE was highly abundant in Aβ plaques in DS and LOAD (Fig. 5B, D) compared to EOAD, being the most significant in DS (Fig. 5B) in comparison to EOAD and LOAD. We also identified a group of glial related proteins in EOAD network, namely VIM, DES and GFAP (Fig. 5C). Overall, our findings suggest a similar plaque protein signature in the three groups, which were functionally associated mainly to APP and Aβ processing, immunity-related responses and lysosomal functions.

**Figure 6:**
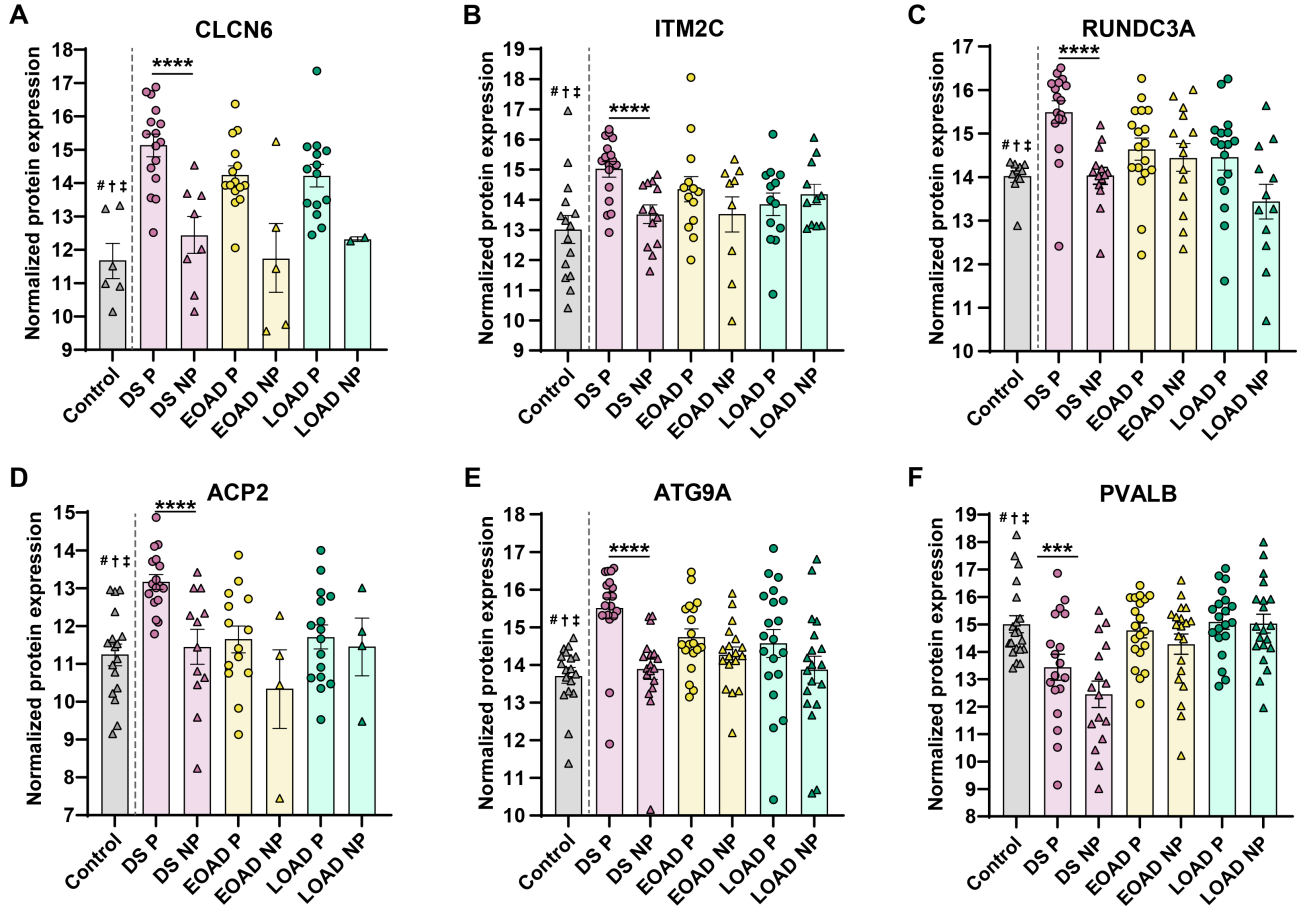
Enriched Aβ plaque proteins of interest in DS compared with EOAD and LOAD. (**A-F**) Normalized protein expression obtained from the label-free quantitative mass spectrometry proteomics of abundant Aβ plaque proteins of interest in DS. Proteins are shown by order of decreasing significance. Proteins of interest were defined as significant (FDR < 5%, fold-change > 1.5) only in DS and also have known or predicted roles in AD and DS. Pairwise comparisons p values are indicated. * *p*<0.05, **** *p*<0.0001. Error bars indicate standard error of the mean (SEM). Significant pairwise comparisons are indicated for those analyses that were performed, controls are shown as reference. # † ‡ indicate that the given protein is not significantly abundant in non-plaque AD tissue compared to controls in DS, EOAD and LOAD, respectively.

In addition, an analysis of the 10 most abundant proteins (ranked by FC) differentially enriched in Aβ plaques in DS, EOAD or LOAD further showed the relationship of Aβ plaque-associated proteins with lysosomal and immune related functions (**Supp. Table 14**). According to the GO annotation, we found that the significantly enriched amyloid plaque proteins in DS predominantly relate to endo/lysosomal functions, including CLCN6, ATG9A and VAMP7 (Fig. 6). We identified protein ITM2C, which is involved in Aβ peptide production (36) (Fig. 6B). We also observed proteins with functions linked to presynaptic signaling and axon guidance, namely RUNDC3A and NTN1 (37, 38) (Fig. 6). The calcium binding protein and marker of inhibitory neurons PVALB was significantly enriched in DS plaques, but was unaltered in EOAD and LOAD (Fig. 6F). In contrast, we observed that Aβ plaque proteins significantly abundant in EOAD are mostly related to immune response, immunoglobulin mediated immune response (S100A7, HPX, IL36G) as well as vacuole lumen and secretory vesicles related (GGH, TTR). The protein EPPK1 is linked to cytoskeletal organization functions such epithelial cell proliferation and intermediate filament organization (**Supp. Table 14**). In LOAD, we observed a series of proteins involved in bounding membrane of organelle, collagen-containing extracellular matrix and vesicle membrane (CYB5B, VWF and PTPRN2). Although we did not observe particular association with GO terms, other amyloid plaque LOAD proteins including TIMM8A, ACSS3 and SFXN5 (linked to mitochondrial functions) (39–41), THUMPD1 and RPS7 (related to RNA binding activity and ribosomes) (42, 43) and NRXN2 (protein-protein interactions at the synapses) (44), were identified (**Supp. Table 14**). These observations support our findings in the GO functional enrichment and protein interaction networks, providing evidence that some of the most abundant proteins in DS plaques are primarily linked to lysosomal pathways.

#### Non-plaque Tissue Functional Analyses

GO terms for abundant non-plaque proteins showed chromatin remodeling as the top BP term for all experimental groups (DS *p* = 0.00128, EOAD *p* = 5.79×10^− 9^, LOAD *p* = 1.69×10^− 10^, **Supp.** Figure 2A, **Supp. Table 8**). Importantly, top BP GO terms in DS were associated with integrin-mediated signaling, extracellular structure and extracellular matrix organization (*p* = 0.00684, **Supp.** Figure 2A, **Supp. Table 8**). In contrast, EOAD and LOAD top BP GO terms included Protein-DNA complex assembly (*p* = 4.74×10^− 6^ and *p* = 1.14×10^− 8^, respectively), regulation of gene expression (EOAD p = 5.08×10^− 5^, LOAD *p* = 1.68×10^−8^) and nucleosome assembly (EOAD *p* = 4.74×10^− 6^, LOAD *p* = 3.25×10^− 8^) (**Supp.** Figure 2A, **Supp. Table 8**). Top CC GO terms for DS were collagen-containing extracellular matrix, which was also observed in EOAD and LOAD, external encapsulating structure and extracellular matrix (*p* = 3.52×10^− 8^, **Supp.** Figure 2A, **Supp. Table 12**). Top CC GO term for EOAD was nucleosome (*p* = 4.44×10^− 6^), which was also identified in DS and LOAD. Other EOAD top CC GO terms were DNA packaging complex (*p* = 8.01×10^− 6^) and protein-DNA complex (*p* = 2.23×10^− 5^) (**Supp/** Figure 2A, **Supp. Table 12**). In a similar fashion, LOAD top CC GO terms were DNA packaging complex, protein-DNA complex (both *p* = 3.78×10^−14^) and nucleosome (*p* = 1.71×10^− 12^) (**Supp.** Figure 2A, **Supp. Table 12**).

We also created protein interaction networks of non-plaque tissue DS, EOAD and LOAD proteomes, which showed a highly significant degree of protein-protein interactions (PPI Enrichment *p* = 1 × 10^− 16^, **Supp.** Figure 2B-D). We observed groups of RNA binding proteins such as SRSF4, eukaryotic initiation factors (eIF4) and the heterogeneous nuclear ribonucleoproteins (hnRNP) protein family, primarily in EOAD and LOAD networks (**Supp.** Figure 2C, D). We also observed a set of intermediate filament and glial proteins such as GFAP, AQP4, DES, VIM, ALDH1L1 and GART (**Supp.** Figure 2B-D). Additionally, there were groups of histone proteins related to the nucleosome, such as H2A, H2B and H1 protein families (**Supp.** Figure 2B-D). Particularly, the DS protein interaction network exhibited a set of collagens, laminins, cell adhesion proteins, proteoglycans and heparin sulfate proteins (**Supp.** Figure 2B) as well as proteasome and chaperone proteins also involved in regulation of gene expression, including SQSTM1, PSMB4, PSMD4 and HSPB6 (**Supp.** Figure 2B). Our findings highlight a pivotal role of extracellular matrix (ECM) and structural components in DS besides the proteins associated to Aβ plaque pathology.

#### Comparative analysis with previous human AD proteomics and identification of novel plaque proteins

**Figure 7:**
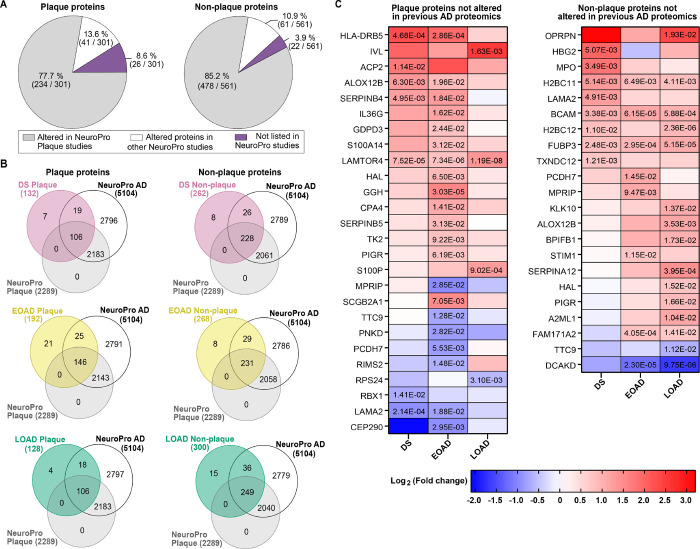
Comparison of protein changes with previous advanced AD proteomics studies. A. Altered proteins identified in the current study were compared with proteins found altered in previous AD proteomics compiled in NeuroPro (35) (v1.12; https://neuropro.biomedical.hosting/). Pie charts show that 77.7% (234/301) of altered plaque proteins in the present study have been identified in previous AD plaque proteomics studies (gray). 13.6% (41/301) of the proteins have been seen only in bulk tissue proteomics studies (white), and 8.6% (26/301) of the altered proteins observed in the current study have not been described in previous AD proteomics (purple). In a similar fashion, 85.2% (478/561) proteins altered in AD non-plaque tissue have been observed in AD plaque proteomics, 10.9% (61/561) only in bulk tissue proteomics and 3.9% (22/561) have not been described in previous AD proteomics studies. B. Venn diagrams illustrate the altered proteins identified in Aβ plaques and AD non-plaque tissue for each AD subtype evaluated, in comparison to the 5104 altered proteins in advanced AD registered in NeuroPro database. C. Heatmaps depicting the fold change (Log2 [FC]) of the plaque and AD non-plaque altered proteins identified in the present study that have not been described in previous AD proteomics. Numbers in the cells represent the significance (FDR < 0.05) values observed in the pairwise comparisons.

We compared the differentially abundant proteins found in Aβ plaques and AD non-plaque tissue with previous human AD proteomics studies compiled in the NeuroPro database (35). We observed that 77.7% of altered proteins identified in amyloid plaques in our study were also identified in previous AD plaque proteomics studies (Fig. 7A). From the 301 significantly altered plaque proteins that we identified in the present study, 13.6% have not been found in previous plaque proteomics studies, but only reported as significantly altered in bulk brain tissue proteomics studies (Fig. 7A). Similarly, 85.2% of the proteins we identified in the non-plaque tissue have been described in previous plaque and bulk tissue proteomics studies, whereas 10.9% have been identified in bulk human brain tissue but not in plaque proteomics studies (Fig. 7A). Interestingly, we identified in our study 34 proteins that have not been described previously in any human AD proteomics study, either in plaques or in bulk tissue (Fig. 7A, **Supp. Table 15–16**).

In DS specifically, we identified seven amyloid plaque proteins and eight non-plaque tissue proteins significantly altered in our study, which have not been found in past AD proteomics studies (Fig. 7B, **Supp. Table 17**). Similarly, we identified in EOAD 21 significantly altered proteins in plaque and eight in non-plaque tissue, which have not been described previously (Fig. 7B, **Supp. Table 17**). In the case of LOAD, we observed four significantly altered proteins in amyloid plaques and 15 in non-plaque tissue that have not been identified in previous AD plaques or bulk brain tissue proteomics studies (Fig. 7B, **Supp. Table 17**). From this group of proteins, LAMTOR4 (late endosomal/lysosomal adaptor and MAPK and MTOR activator 4) was significantly enriched in Aβ plaques in all the cohorts analyzed (Fig. 7C). The proteins HLA-DRB5, ALOX12B and SERPINB4 were significantly enriched in DS and EOAD amyloid plaques (Fig. 7C). In contrast, LAMA2 was significantly decreased in DS and EOAD amyloid plaques (Fig. 7C). On the other hand, we observed the histone protein H2BC11, the basal cell adhesion protein BCAM and the DNA binding protein FUBP3 significantly enriched in non-plaque tissue in DS, EOAD and LOAD (Fig. 7C). The protein FAM171A2 was significantly enriched only in EOAD and LOAD, contrary to the protein DCAKD that was significantly decreased in EOAD and LOAD non-plaque tissue (Fig. 7C). Overall, our proteomics findings are consistent with previous proteomics studies. Notably our comparative analysis allowed us to identify novel proteins in AD human proteomics.

#### Validation of the Aβ plaques protein signature in DS and novel plaque proteins in human DS proteomics

**Figure 8:**
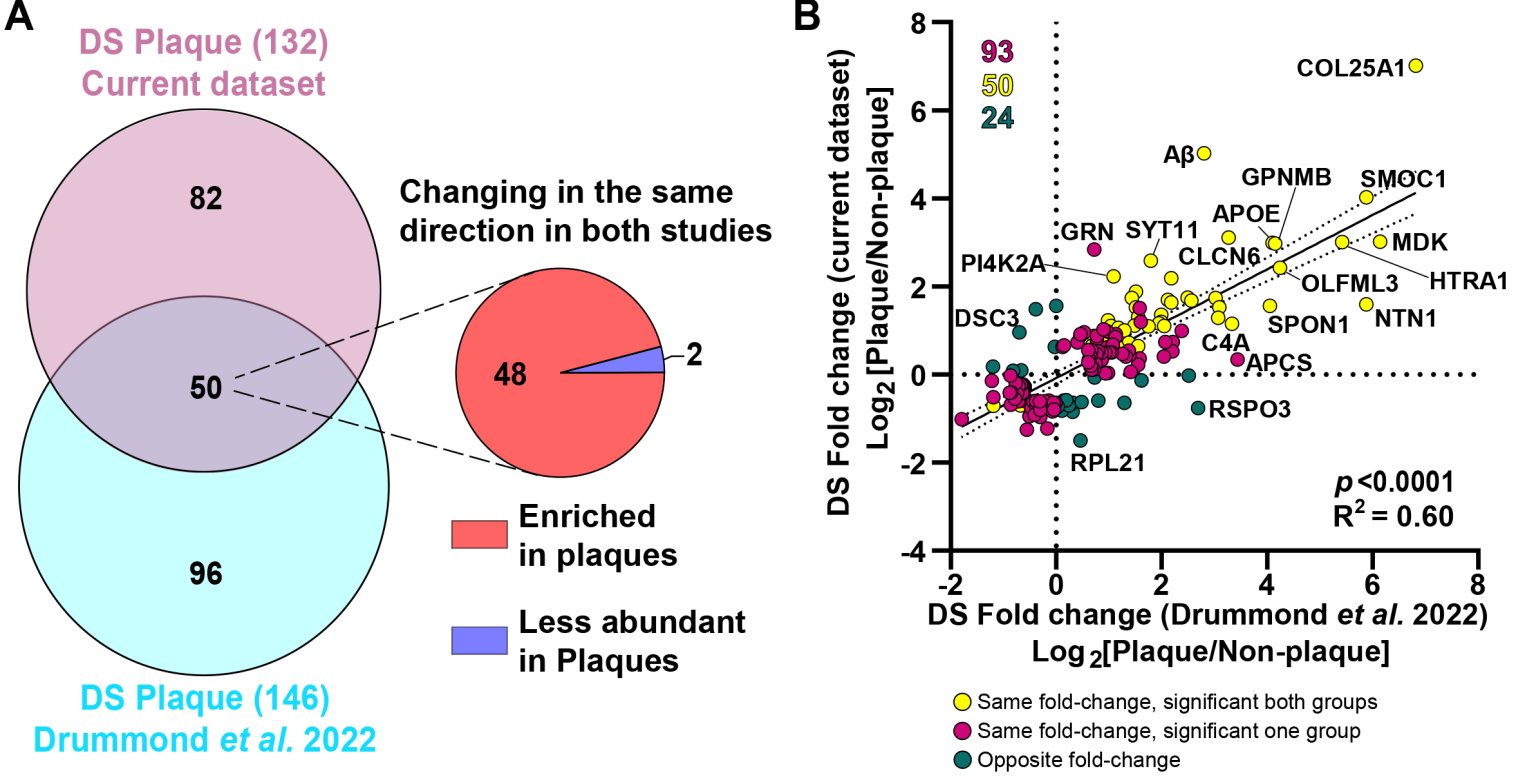
Comparison of protein changes between the DS plaques localized proteomics studies. **A.** Venn diagram depicts differentially abundant proteins identified in the current study and the previous DS plaque proteomics study (Drummond *et al*. 2022, (12)). We identified 132 significantly altered proteins compared to 146 identified previously. From the 50 common proteins identified, 48 were enriched in Aβ plaques and 2 proteins were less abundant in both studies. **B.** Correlation analysis between differentially abundant proteins in the current study and previous DS localized proteomics. Yellow dots represent significant proteins changing in the same direction (highly abundant or less abundant proteins in both groups evaluated) in both groups compared. Magenta dots represent proteins changing in the same direction, but are significant only in one of the groups evaluated. Green dots represent proteins changing in opposite direction (i.e., abundant in one group and less abundant in the other group evaluated). There was a significant positive correlation (*p* < 0.0001, R^2^=0.60) between the two datasets.

The NeuroPro database is a powerful tool to investigate proteomic changes in AD human brains. However, the database does not include DS proteomics data. Therefore, we compared our DS amyloid plaques proteomics findings with our previous study (Drummond *et al*., 2022 (12)) where unbiased localized proteomics was used to interrogate the DS amyloid plaques proteome. We observed 2522 proteins between both DS plaque proteomics datasets, comprised of 1981 proteins in the present study and 2258 proteins in our previous work (excluding isoforms). We observed 68.1% (1717/2522) of proteins overlapping between both studies. We also identified 228 significantly altered plaque proteins between both studies. We observed that 21.9% (50/228) of proteins were common (Fig. 8A). In addition, 36% (82/228) of the proteins significantly altered in the present study were not significant in Drummond et al. study (Fig. 8A, **Supp. Table 18**). In contrast, 42.1% (96/228) of the proteins identified by Drummond *et al*. were not detected in the current dataset (Fig. 8A, **Supp. Table 18**). Despite the proteins differences on each study, we observed a significant positive correlation between the amyloid plaque proteomes of the DS cohorts (*p* < 0.0001, R^2^ = 0.60, Fig. 8B). In fact, the 50 common proteins between both studies were changing in the same direction (48 enriched and 2 decreased in plaques, Fig. 8B). Within these set of amyloid plaque proteins we identified Aβ peptide, APP, COL25A1, and a set of previously described plaque proteins such as APOE, SMOC1, CLU, C3, CLCN6 among others (extended data in **Supp. Table 18**), thus validating a plaque protein signature also observed in DS Aβ pathology. Interestingly, from the seven novel DS plaque proteins regarding the NeuroPro database (**Supp. Table 17**), only ACP2 was also observed in the previous DS plaque proteomics study (**Supp. Table 18**). Our study is consistent with previous similar proteomics studies on AD brains, and further expanded the proteins present at these pathological lesions.

## Discussion

We conducted an extensive comparative analysis of the Aβ plaque and non-plaque proteomes in individuals with DS, EOAD, and LOAD. We identified 43 proteins consistently altered in Aβ plaques throughout all cohorts. The Aβ plaque proteomes showed a high correlation across AD subtypes, with some proteins exhibiting differential abundance in each group. GO functional enrichment and protein-protein interaction analyses indicated a predominant association of Aβ plaque proteins with APP metabolism, lysosomal functions, and immune response. Our results suggest a similar ‘Aβ plaque protein signature’ across the groups evaluated, highlighting a significant similarity between the DS plaque proteome and those of EOAD and LOAD. In contrast, the non-plaque proteome showed variations in protein abundance among the groups evaluated, resulting in different functional associations. Our results highlight physiological alterations in the brains of individuals with DS in relation to EOAD and LOAD.

Our unbiased localized proteomics allowed us to identify hundreds of proteins associated with Aβ plaques, including HTRA1, CLU, CLSTN1, GPC1, and VIM, which have been linked to protective roles against Aβ neuropathology or as regulators of amyloid production (45–48). We also confirmed the presence of less studied proteins in AD in Aβ plaques, such as CLCN6, ARL8B, TPP1, VAMP7, and SMOC1 (12). These results underscore the potential role of less studied proteins in AD neuropathology. Additionally, our comparison with earlier studies led us to identify several plaque enriched proteins not previously reported in human AD proteomics or DS proteomics. These novel plaque proteins are associated with crucial processes in Alzheimer's neuropathology and DS including lysosomal functions (ACP2, LAMTOR4), immune response (HLA-DRB5, IL36G), and ubiquitination (RBX1) (49–55). Notably, these proteins have been linked to Alzheimer's disease solely through genetic studies. Therefore, we have expanded on these previous findings, showing a probable association between these proteins and AD pathophysiology.

As shown in our protein network analysis, we observed a functional pattern among the plaque proteins with a higher degree of predicted protein-protein interactions in all experimental groups. For instance, we observed the plaque proteins NTN1, NCSTN, SPON1, and CLSTN1 in all cohorts, which have been related to APP/Aβ processing (48, 56–64). The role of APP metabolism in AD has long been recognized, with the APP gene mapped to chromosome 21 (65). However, these proteins associated with APP remain understudied in DS. Our proteomics analysis revealed the presence of immune and inflammation-related proteins, including C1QC, C4A, C3, MDK, CLU, HLA-DRB1, and HLA-DRB5. Notably, these proteins formed clusters adjacent to the APP node in the protein networks, suggesting potential interactions with Aβ. This finding is consistent with earlier research associating complement proteins, CLU, and MDK with senile plaques (16, 66). Specifically, murine studies have demonstrated that CLU contributes to neurotoxicity and the deposition of fibrillary Aβ (67). Conversely, MDK has been shown to bind Aβ, with transgenic mouse studies indicating a reduction in Aβ deposition, although the underlying mechanisms remain unclear (68). Furthermore, studies using mouse models of AD have indicated that complement system proteins play a role in synapse loss, the formation of dystrophic neurites, and increased Aβ aggregation, possibly through microglia-astrocyte crosstalk in response to amyloid pathology (as reviewed by Batista and colleagues (69), (70–73)). Our proteomics findings highlighted the enrichment in Aβ plaques of the proteins HLA-DRB1 and the novel plaque protein HLA-DRB5. Previous single-cell transcriptomics studies using human AD prefrontal cortex observed correlation of *HLA-DRB1* and *HLA-DRB5* expression in microglia with measures of AD pathology (74, 75). Notably, HLA proteins mechanisms in Aβ neuropathology remain unknown.

Our Aβ plaques proteomics data also highlighted the enrichment of multiple proteins related to the endo/lysosomal pathway, providing additional support to previous findings describing lysosomal dysfunction as one of the earliest events upregulated in AD (76–79). We identified TPP1, PPT1, LAMP1, ARL8B and VAMP7, which are involved in lysosomal trafficking, vesicle fusion and degradation processes within the lysosomes (80–82). Deficiency of TPP1 and PPT1 have been linked to the neurodegenerative lysosomal storage disease neuronal ceroid lipofuscinosis (NCL) (83), and ARL8B also has been linked to a lysosomal storage disorder, Niemann-Pick disease type C (84). LAMP1 involvement in amyloid pathology has been widely acknowledged, found in reactive microglial cells in senile plaques rather than diffuse deposits, suggesting an involvement in amyloid removal (85). TPP1 has been reported to destabilize Aβ by endoproteolytic cleavages (86), whereas ARL8B has been related to a neuroprotective effect against amyloid pathology (87). Importantly, our data confirmed the protein VAMP7, identified as a novel amyloid plaque protein in our previous study (12).

A closer examination of the most significant functional associations in the DS Aβ plaque proteome elucidated a substantial enrichment of lysosomal-related GO terms, followed by those linked to the immune system and cell activation. Both lysosomal and immune processes are integral to AD pathophysiology (16, 77, 78, 88–91). Strong evidence suggests that endo/lysosomal alterations in DS are associated with APP and the βCTF fragment produced after BACE-1 cleavage of APP, potentially explaining early changes in DS (92–95). Increased systemic inflammation, possibly exacerbated by Aβ accumulation, is also evident in individuals with DS (96, 97). Interestingly, the functional associations observed in the DS plaque proteome appear to be a combination of those found in EOAD and LOAD, further highlighting the Aβ plaque proteome similarity across cohorts.

Significant plaque proteins consistently demonstrated enrichment across all cohorts, yet certain proteins were distinctly enriched in specific experimental groups. This observation may be crucial for understanding the pathogenesis of AD and illuminating the unique mechanisms driving the disease in DS and various AD subtypes. A case in point is COL25A1, also known as CLAC-P, which was the most enriched protein in plaques. Interestingly, the abundance of COL25A1 in DS plaques surpassed that in EOAD and LOAD plaques. Prior studies in murine models suggested that CLAC, a derivative of COL25A1, plays a pivotal role in converting diffuse Aβ deposits into senile plaques. (98, 99). This finding may partially account for the heightened amyloid pathology observed in DS. Moreover, previous research has shown that the interaction between CLAC and Aβ is determined by negatively charged residues in the central region (100). Given recent discoveries about Aβ filaments in DS and Aβ fibril variation in different AD subtypes, structural differences in Aβ fibrils may result in unique interactions with COL25A1 (101, 102). Further investigation is required to comprehend the binding affinity of COL25A1 in DS and other forms of AD. However, our previous study indicated similar levels of COL25A1 in DS and EOAD plaques (12). It is plausible that the observed differences between our current and past studies are due to technical factors such as sample preparation, data acquisition, and cohort size (103).

Our proteomics analysis also revealed a significant reduction of the protein HAPLN2 within Aβ plaques across all three cohorts evaluated. This protein, primarily expressed in oligodendrocytes, plays a crucial role in stabilizing the extracellular matrix components at the nodes of Ranvier (104, 105). Interestingly, we also observed a decrease in several other oligodendrocyte proteins, including PLP1, MOG, MAG, MBP and BCAS1. Furthermore, we noted a reduction in oligodendrocyte proteins in the non-plaque proteome compared to controls. The protein PLP1 was significantly decreased in DS and EOAD, while MOG, MAG, and MBP were notably reduced in DS compared to EOAD and LOAD. These findings align with previous research on the role of myelin degeneration in AD pathogenesis and suggests a more significant impact on DS. A study in rhesus monkeys of different ages linked myelin degeneration to normal aging and cognitive decline (106). Recent studies using transgenic mice and human AD tissues have shown that myelin defects, directly and indirectly, promote Aβ plaque formation, alongside transcriptional changes in oligodendrocytes seen in AD and other degenerative diseases (107, 108). Given that individuals with DS often exhibit age-associated disorders earlier than euploid individuals (109), it is plausible that myelin damage is an early characteristic in DS, potentially exacerbating amyloid pathology. Further studies are needed to elucidate how oligodendrocytes are impacted in DS and AD.

The analysis of the non-plaque tissue proteome in DS, EOAD, and LOAD highlighted two primary altered components in AD: the ECM and chromatin structure. Specifically, within the protein networks of the DS non-plaque proteome, we observed a cluster of proteins related to ECM, which was not evident in the networks of EOAD and LOAD but suggested by functional annotation analysis. Early studies using human AD brain samples provided evidence of ECM proteins (namely collagen, laminin and HSPG) colocalizing with neuritic plaques (110). Subsequent findings in transgenic mice and human AD brain samples showed increased mRNA levels of collagen type VI proteins in a dose-dependent proportion to the expression of APP and Aβ, also suggesting potential protective roles of this collagen against Aβ neurotoxicity (111). This evidence aligns with our observations in all cohorts. However, our data indicate that the ECM in DS is more significantly affected compared to EOAD and LOAD. More recent studies using trisomy 21 induced pluripotent stem cells (iPSCs) suggested aberrant ECM pathways and increased cell-cell adhesion, which may lead to reduced proliferation and migration, thus affecting neural development (112, 113). In addition, proteomics studies of human AD brain tissues indicated a correlation of cell-ECM interaction pathways and matrisome components with AD neuropathological and cognitive traits (114). The authors also observed ECM components in pre-clinical AD cases, suggesting that ECM might be altered in early stages of AD. These observations support a more significant and earlier alteration of ECM proteins in DS, possibly exacerbated by AD neuropathology. Moreover, proteins linked to chromatin structure were consistently altered in non-plaque tissue in all groups studied, most prominently in LOAD and EOAD. Our observations align with previous research suggesting structural changes in chromatin accessibility and consequent altered gene expression in AD (115–118). Studies using murine models of DS and trisomy 21 iPSCs have elucidated reduced global transcription activity and changes resembling those observed in senescent cells such as ‘chromosomal introversion’, disruption of the nuclear lamina, and altered chromatin accessibility (119, 120). This evidence may explain the differences we observed in the protein interaction networks and functional annotation analyses between the non-plaque proteomes of DS and the studied AD subtypes.

While our study sheds light on the molecular mechanisms behind Aβ plaque pathology in DS and various forms of AD, it is essential to recognize certain limitations. We restricted our analysis to classic cored plaques and dense aggregates from DS and AD cases primarily at advanced disease stages, constraining our conclusions to an ‘end-point’ proteome profile. Nonetheless, we identified notable neuropathological distinctions between DS and other cohorts, potentially associated with observed proteomic alterations in plaque and non-plaque tissues. Future studies targeting different morphological types of plaques (i.e., diffuse or cotton-wool plaques) would be interesting. Our analysis was also limited to vulnerable brain regions in AD. Future investigations should encompass broader age ranges and additional brain regions, including those resistant to AD, to enhance comprehension of disease progression and resilience mechanisms. Furthermore, membrane proteins, particularly integral membrane proteins, are often underrepresented in proteomics studies due to detection challenges. Lastly, while our research is unbiased, it remains susceptible to variability arising from unknown genetic factors in each case. Subsequent research endeavors should integrate genetic details, such as familial AD mutations and other known genetic variables, to gain deeper insights into their impact on AD.

## Conclusions

Our study offers novel insights into the amyloid plaque proteome of DS, elucidating key functional aspects underlying the disease and contrasting them with those of EOAD and LOAD. We have demonstrated a notable similarity among the plaque proteomes of DS, EOAD and LOAD, highlighting the predominant functional associations of plaque proteins with endo/lysosomal pathways, immunity, and APP metabolism. Through the analysis of the non-plaque proteome, we have provided significant findings about the differential alteration of ECM and chromatin structure, highlighting nuanced differences between DS, EOAD and LOAD. Our unbiased proteomics approach not only identifies enriched plaque proteins but also suggests potential therapeutic targets or biomarkers for AD, thereby offering promising avenues for future research and clinical applications.

## Figures and Tables

**Table 1:** Case history summary.

Group	Cases	Mean Age at Death (years)*	Sex	Mean PMI (hours)*	Neuropathology	*APOE* genotype
Down syndrome	20	59.8 ± 4.99	7 F / 13 M	17.95 ± 11.71	Equivalent to A3, B3, C3 score or Braak V-VI, Thal 5	*ε*3/*ε*3: 13, *ε*4/*ε*4: 2, *ε*3/*ε*4: 3, *ε*2/*ε*4: 1
EOAD	20	63 ± 4.07	5 F / 15 M	27.47 ± 12.76	Equivalent to A3, B3, C3 score or Braak V-VI, Thal 4	*ε*3/*ε*3: 10, *ε*4/*ε*4: 3, *ε*3/*ε*4: 5, *ε*2/*ε*3: 2
LOAD	20	^#^ 82.1 ± 6.37	10 F / 10 M	33.22 ± 19.19	A3, B3, C3 or Braak VI	*ε*3/*ε*3: 6, *ε*4/*ε*4: 3, *ε*3/*ε*4: 7, *ε*2/*ε*3: 2, *ε*2/*ε*4: 2
Control	20	66.4 ± 13.04	9 F / 11 M	59.50 ± 27.30	≤ A1, B1, C1	N/A

* Mean Age at death and Mean PMI ± Standard deviation.

# Significant differences by one-way ANOVA.

**Table 2:** Top 20 signifi cant proteins in Down syndrome, early-onset and late-onset AD for ‘plaque vs. non-plaque’ pairwise comparisons.

Down syndrome - Plaque vs Non-plaque						
Uniprot Accession ID	Gene name	Name	p-value	Fold Change	Change in EOAD	Change in LOAD
***Increased***						
Q9BXS0	COL25A1	Collagen alpha-1(XXV) chain	2.51E-12	129.5	↑	↑
	Aβ		8.16E-09	32.5	↑	↑
Q92743	HTRA1	Serine protease HTRA1	2.24E-09	8.1	↑	↑
P02649	APOE	Apolipoprotein E	8.6E-13	8.0	↑	↑
O94985	CLSTN1	Calsyntenin-1	4.12E-12	3.3	↑	↑
P05067	APP	Amyloid-beta precursor protein	1.07E-09	3.2	↑	↑
P35052	GPC1	Glypican-1	9.46E-09	2.9	↑	↑
P10909	CLU	Clusterin	7.95E-09	2.6	↑	↑
O14558	HSPB6	Heat shock protein beta-6	7.59E-10	1.9	↑	↑
P08670	VIM	Vimentin	6.01E-09	1.8	↑	↑
**Decreased**						
P0DP58	LYNX1	Ly-6/neurotoxin-like protein 1	5.39E-06	3.3	↓	↓
P42677	RPS27	40S ribosomal protein S27	4.11E-05	1.9	↓	
Q9GZV7	HAPLN2	Hyaluronan and proteoglycan link protein 2	3E-06	1.9	↓	↓
P10915	HAPLN1	Hyaluronan and proteoglycan link protein 1	2.03E-07	1.9	↓	↓
P62942	FKBP1A	Peptidyl-prolyl cis-trans isomerase FKBP1A	1.26E-05	1.9	↓	
Q8WY54	PPM1E	Protein phosphatase 1E	7.22E-06	1.8		↓
P13987	CD59	CD59 glycoprotein	4.05E-05	1.8		↓
Q8NCB2	CAMKV	CaM kinase-like vesicle-associated protein	4.01E-06	1.6		
O75363	BCAS1	Breast carcinoma-amplified sequence 1	1.48E-05	1.5	↓	↓
Q9H9H5	MAP6D1	MAP6 domain-containing protein 1	2.36E-05	1.5	↓	
Early-onset AD - Plaque vs Non-plaque						
Uniprot Accession ID	Gene name	Name	p-value	Fold Change	Change in DS	Change in LOAD
**Increased**						
	Aβ		6.43E-10	21.6	↑	↑
Q92743	HTRA1	Serine protease HTRA1	1.84E-08	6.0	↑	↑
P02649	APOE	Apolipoprotein E	3.18E-10	5.9	↑	↑
Q9BT88	SYT11	Synaptotagmin-11	3.45E-09	2.9	↑	↑
P35052	GPC1	Glypican-1	1.51E-09	2.6	↑	↑
O94985	CLSTN1	Calsyntenin-1	9.36E-10	2.5	↑	↑
P0C0L4	C4A	Complement C4-A	5.49E-08	2.4	↑	↑
P08670	VIM	Vimentin	7.4E-10	2.1	↑	↑
P07339	CTSD	Cathepsin D	1.97E-09	2.0	↑	↑
P26038	MSN	Moesin	5.16E-08	1.7		↑
**Decreased**						
O94772	LY6H	Lymphocyte antigen 6H	2.55E-06	2.2		↓
Q9GZV7	HAPLN2	Hyaluronan and proteoglycan link protein 2	2.88E-08	1.9	↓	↓
Q16653	MOG	Myelin-oligodendrocyte glycoprotein	5.84E-07	1.9	↓	↓
P60201	PLP1	Myelin proteolipid protein	1.18E-06	1.9	↓	↓
Q7Z3B1	NEGR1	Neuronal growth regulator 1	5.09E-07	1.8		
P09543	CNP	2',3'-cyclic-nucleotide 3'-phosphodiesterase	4.73E-09	1.7		
P02686	MBP	Myelin basic protein	1.97E-06	1.7		↓
P13637	ATP1A3	Sodium/potassium-transporting ATPase subunit alpha-3	1.95E-09	1.6		↓
P11169	SLC2A3	Solute carrier family 2, facilitated glucose transporter member 3	1.97E-06	1.5		↓
P41594	GRM5	Metabotropic glutamate receptor 5	1.45E-07	1.5		↓
Late-onset AD - Plaque vs Non-plaque						
Uniprot Accession ID	Gene name	Name	p-value	Fold Change	Change in DS	Change in EOAD
**Increased**						
	Aβ		2.55E-09	25.8	↑	↑
Q92743	HTRA1	Serine protease HTRA1	9.94E-09	6.2	↑	↑
P35052	GPC1	Glypican-1	1.39E-09	3.2	↑	↑
Q9BT88	SYT11	Synaptotagmin-11	1.5E-09	2.9	↑	↑
Q0VGL1	LAMTOR4	Ragulator complex protein LAMTOR4	1.19E-08	2.5	↑	↑
P14136	GFAP	Glial fibrillary acidic protein	2.78E-09	2.4		↑
P08670	VIM	Vimentin	1.87E-10	2.4	↑	↑
Q9ULB1	NRXN1	Neurexin-1	4.05E-08	2.4	↑	↑
Q9UM22	EPDR1	Mammalian ependymin-related protein 1	4.23E-08	1.9	↑	↑
P55084	HADHB	Trifunctional enzyme subunit beta, mitochondrial	4.83E-08	1.5		
**Decreased**						
Q6UWR7	ENPP6	Glycerophosphocholine cholinephosphodiesterase ENPP6	1.23E-06	2.0	↓	↓
O75363	BCAS1	Breast carcinoma-amplified sequence 1	9.47E-06	1.8	↓	↓
Q9GZV7	HAPLN2	Hyaluronan and proteoglycan link protein 2	5.75E-06	1.7	↓	↓
Q8IXJ6	SIRT2	NAD-dependent protein deacetylase sirtuin-2	7.07E-07	1.7		↓
P60201	PLP1	Myelin proteolipid protein	3.38E-07	1.6	↓	↓
Q16653	MOG	Myelin-oligodendrocyte glycoprotein	1.25E-06	1.6	↓	↓
P20916	MAG	Myelin-associated glycoprotein	4.16E-06	1.6		↓
P02686	MBP	Myelin basic protein	7.42E-06	1.6		↓
P11169	SLC2A3	Solute carrier family 2, facilitated glucose transporter member 3	3.52E-05	1.5		↓
P13637	ATP1A3	Sodium/potassium-transporting ATPase subunit alpha-3	2.8E-07	1.5		↓

## Data Availability

The resulting mass spectrometry raw data are accessible on the MassIVE server (https://massive.ucsd.edu/) under MassIVE ID: MSV000094800. All data analyzed during this study is included in this published article and its supplementary information files.

## Abbreviations

DS: Down syndrome
Hsa21: Human chromosome 21
AD: Alzheimer’s disease
APP: Amyloid precursor protein
Aβ: Amyloid-β
EOAD: early-onset Alzheimer’s disease
LOAD: late-onset Alzheimer’s disease
ECM: Extracellular matrix
FFPE: Formalin-fixed and paraffin-embedded
LCM: Laser-capture microdissection
LC-MS: Liquid chromatography–mass spectrometry
FDR: False discovery rate
PCA: Principal component analysis
GO: Gene ontology
BP: Biological process
CC: Cellular component
PPI: Protein-protein interaction networks
